# Gentamicin loaded niosomes against intracellular uropathogenic *Escherichia coli* strains

**DOI:** 10.1038/s41598-024-59144-x

**Published:** 2024-05-03

**Authors:** Jacopo Forte, Linda Maurizi, Maria Gioia Fabiano, Antonietta Lucia Conte, Maria Pia Conte, Maria Grazia Ammendolia, Eleonora D’Intino, Angela Catizone, Luisa Gesualdi, Federica Rinaldi, Maria Carafa, Carlotta Marianecci, Catia Longhi

**Affiliations:** 1 Dipartimento di Chimica e Tecnologie del Farmaco, Sapienza Università di Roma, Piazzale Aldo Moro, 5, 00185 Rome, Italy; 2 Dipartimento di Sanità Pubblica e Malattie Infettive, Sapienza Università di Roma, Piazzale Aldo Moro, 5, 00185 Rome, Italy; 3 Centro Nazionale Tecnologie Innovative in Sanità Pubblica, Istituto Superiore di Sanità, Viale Regina Elena, 299, 00161 Rome, Italy; 4 Dipartimento Scienze Anatomiche, Istologiche, Medico Legali e Dell’Apparato Locomotore, Sapienza Università di Roma, Via Scarpa, 16, 00161 Rome, Italy

**Keywords:** Microbiology, Diseases, Health care, Urology, Nanoscience and technology

## Abstract

Urinary tract infections (UTIs) are the most common bacterial infections and uropathogenic *Escherichia coli* (UPEC) is the main etiological agent of UTIs. UPEC can persist in bladder cells protected by immunological defenses and antibiotics and intracellular behavior leads to difficulty in eradicating the infection. The aim of this paper is to design, prepare and characterize surfactant-based nanocarriers (niosomes) able to entrap antimicrobial drug and potentially to delivery and release antibiotics into UPEC-infected cells. In order to validate the proposed drug delivery system, gentamicin, was chosen as “active model drug” due to its poor cellular penetration. The niosomes physical–chemical characterization was performed combining different techniques: Dynamic Light Scattering Fluorescence Spectroscopy, Transmission Electron Microscopy. Empty and loaded niosomes were characterized in terms of size, ζ-potential, bilayer features and stability. Moreover, Gentamicin entrapped amount was evaluated, and the release study was also carried out. In addition, the effect of empty and loaded niosomes was studied on the invasion ability of UPEC strains in T24 bladder cell monolayers by Gentamicin Protection Assay and Confocal Microscopy. The observed decrease in UPEC invasion rate leads us to hypothesize a release of antibiotic from niosomes inside the cells. The optimization of the proposed drug delivery system could represent a promising strategy to significatively enhance the internalization of antimicrobial drugs.

## Introduction

The intracellular behavior of some pathogens makes it difficult to fight and eradicate the infections. The intracellular environment protects pathogens from the immune system and drugs, some of which cannot pass cell membranes. Uropathogenic Escherichia (UPEC) coli is able to establish a persistent infection in urinary bladder after an initial colonization^1^. UPEC is the major causative agent of UTIs because of the ability to colonize the urethra, bladder, and kidney. Infections of the urethra are usually acute and can be successfully treated with antibiotics, whereas those of the bladder and kidney may progress to be persistent and chronic with serious sequelae^1,2^. Studies employing mouse models showed the ability of UPEC to adhere to and invade host bladder epithelial cells^3^. After UPEC binding to urothelial cells, a Toll-like receptor 4 signaling pathway initiates a cascade of events leading to bacterial internalization^4^. The infection may then follow several pathways, such as the expulsion of UPEC into the urinary lumen via the fusiform vesicles (FVs) or bacterial escape from FVs into the cytosol and establishment of the biofilm-like intracellular bacterial communities (IBCs) within the superficial cell with the ability to re-initiate the infection cycle in adjacent cells. Furthermore, UPEC, sequestered in the phagosomes, can escape from lysosome digestion by neutralizing pH resulting in bacterial expulsion^5^ or entry into autophagosomes where bacteria form quiescent intracellular reservoirs (QIRs)^6^. It is known that quiescent intracellular UPEC can persist long-term in the absence of clinical symptoms and re-emerge to cause recurrent UTIs even when antibiotics are used^6^. The failure of conventional antibiotics to contain intracellular infections is related also to the intrinsic drug characteristics and the intracellular compartments^7^. Poor penetration, inactivation in specific intracellular compartments, limited accumulation in the desired cellular compartments could represent critical features for antibiotic intracellular activity^8,9^. Antibiotics used for UPEC infections are typically β-lactam antibiotics, fluoroquinolones, trimethoprim-sulfamethoxazole and aminoglycosides^10–13^. Among aminoglycosides, gentamicin (GM) is used for the treatment of many types of bacterial infection, especially those involving Gram-negative bacteria but the limit for its use is due to the well-known toxicity. The main adverse effect of aminoglycosides is ototoxicity, neuromuscular blockade and nephrotoxicity^14^. To overcome systemic toxicity, some authors suggested the intravesical instillation of GM for treating recurrent UTIs and lower urinary tract infections in patients with neurogenic bladders, even though this therapy is still off-label with undefined dose, schedule, and value^15–17^. It must also be considered that due to its high hydrophilicity, GM diffuses very slowly and poorly through cell membranes. Due to its weakly basic properties, after reaching the interior of the cell, GM is confined within the lysosomes, where the acidic pH may suppress its activity^18^. The main challenge for intracellular chemotherapy is to design and develop innovative carrier systems for antibiotics capable of releasing the drug inside the cells. Encapsulation of aminoglycoside antibiotics, such as GM, has been attempted by several groups as a means of altering the biodistribution of the drug and reducing its toxicity^19–23^.

Niosomes (Nio) are vesicular nanocarriers able to prevent degradation of loaded active compounds, to improve drug stability, solubility, and bioavailability, to enhance drug bioavailability to site of action. They are characterized by biocompatibility, biodegradability, and a significant tolerability. Niosomes are composed of non-ionic surfactants and nowadays they are gaining increasing interest as drug delivery systems for therapeutic applications^24^. Moreover, surfactants are characterized by penetration enhancer properties and, those employed in this work (Tween 85 and Span 80) are composed of oleic acid moieties which could be useful to inhibit endothelial cell activation and to reduce expression of inflammatory molecules during infection^25^. Since the intravesical route is now demonstrating promise in the treatment of recurrent UTIs^26^ GM-Nio intravesical administration could be used to obtain a local antibacterial treatment.

The aim of this paper is to design, prepare and characterize niosomes able to load, deliver and release GM. Empty and loaded niosomes were prepared and characterized in terms of hydrodynamic diameter, ζ-potential, storage stability in different media, entrapment efficiency and GM release. Cell uptake and the effect of empty and GM-loaded niosomes on the invasion ability of UPEC strains in T24 bladder cell monolayers were also evaluated. The failure of antibiotic therapy due to the UPEC persistence in UTIs pose a growing urgence to seek feasible strategies to counteract the intracellular bacterial lifestyle. The observed antibacterial activity of our GM-carried niosome preparations suggests that this system could be optimized to be used for local administration, i.e., by intravesical route, to obtain a drug bioavailability improvement at the target site and systemic side effect reduction.

## Results

### Physical–chemical characterization of niosomes formulations

A deep physical–chemical characterization of empty and loaded niosomes has been carried out in terms of hydrodynamic diameter, ζ-Potential and PDI. These parameters have been evaluated in two different media: Hepes buffer and Artificial Urine (AUM). Moreover, for the GM-Nio sample, the drug entrapment amount (mg/ml) has been evaluated. The obtained results are shown in Table 1. It is possible to observe that GM-Nio was characterized by an increased Hydrodynamic Diameter with respect to the empty ones while the PDI decreases with the drug inclusion in the vesicles (from 0.28 to 0.20 for Nio and GM-Nio, respectively). Quite the opposite, the ζ-Potential values are negative and similar for both formulations. The entrapped drug amount for GM-Nio was 1 mg/ml.

**Table 1:** Physicochemical features of niosomal formulations in Hepes Buffer and artificial urine medium: vesicle dimensions (Hydrodynamic Diameter), ζ-Potential, polydispersity index (PDI), and concentration of entrapped gentamicin in niosomes (GM-entrapment in mg/ml)

Sample	Hydrodynamic Diameter (nm) ± SD		ζ-Potential (mV) ± SD		PDI ± SD		GM-entrapment (mg/ml)	
	Hepes buffer	AUM	Hepes buffer	AUM	Hepes buffer	AUM	Hepes buffer	AUM
Nio	101.60 ± 3.20	111.40 ± 1.20	-35.60 ± 2.10	-9.80 ± 0.12	0.28 ± 0.02	0.26 ± 0.01	–	–
GM-Nio	211.10 ± 3.50	196.10 ± 1.10	-37.10 ± 1.40	-10.90 ± 2.12	0.20 ± 0.01	0.17 ± 0.03	1.00 ± 0.01	1.00 ± 0.01

Data was obtained as the means of three independent experiments. Errors are the standard deviations (SD) of data.

**Figure 1:**
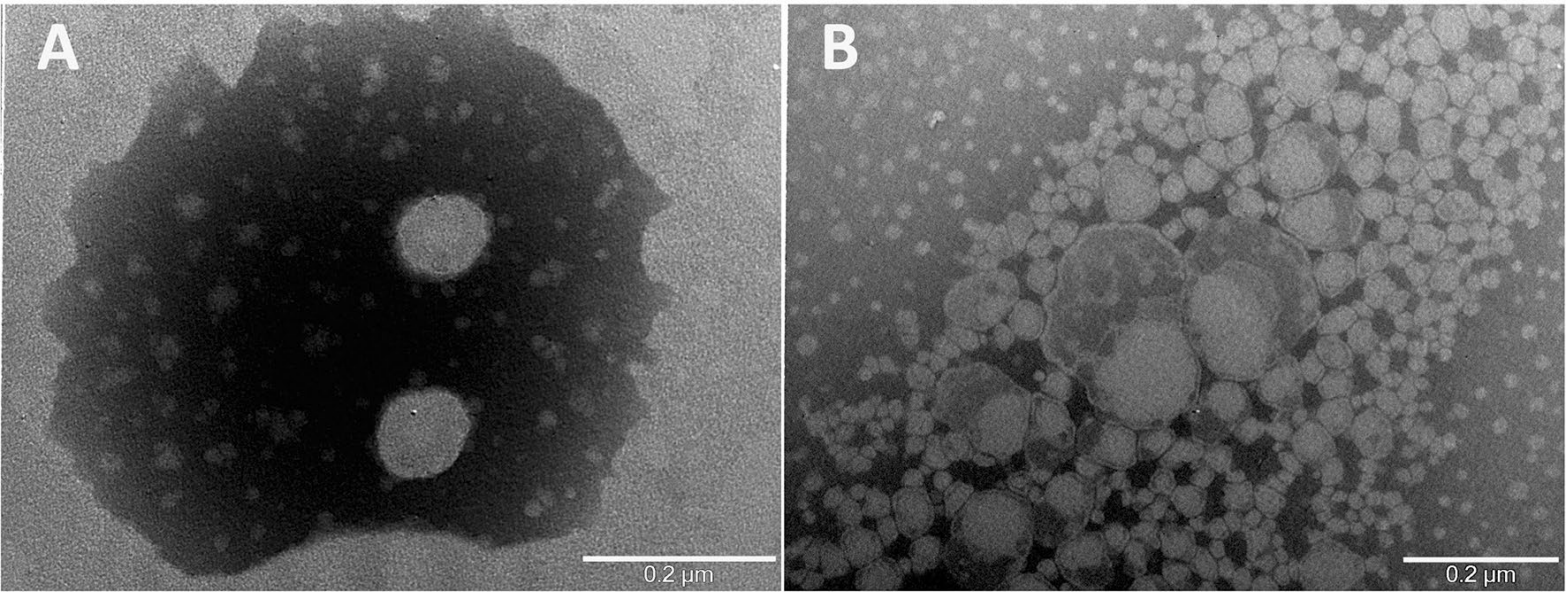
Transmission electron microscopy images of empty (**A**) and GM-loaded niosomes (**B**).

Moreover, empty and GM-Nio vesicles were characterized by ultrastructural analysis. Morphological visualization of empty niosomes showed vesicles almost spherical in shape with unilamellar structure (Fig. 1, panel A). GM loading led to an increase in size, as also observed by DLS measurements. Moreover, GM-loaded niosomes appeared slightly modified in shape with spherical and irregular forms. Unilamellar surface was maintained whereas internal structure appeared inhomogeneous with more electron dense parts, perhaps due to the antibiotic filling (Fig. 1, panel B).

In order to study if the artificial biological fluids affected the niosomal integrity of empty and loaded vesicles, stability study was performed in artificial urine and in RPMI, a medium used in all experiments with cell culture.

The physical–chemical features of both samples, reported in Table 1, were maintained constant in presence of AUM (Table 1) except the ζ-Potential value that became more positive with respect to the samples prepared in Hepes buffer.

In addition, the bilayer features of both formulations have been studied in the same media (Table 2). The GM encapsulation in niosomal formulations caused an evident perturbation of the bilayer, highlighted by the significant increase of anisotropy and polarity values and by the microviscosity decreasing in the GM-Nio with respect to empty ones. However, the bilayer features have been also studied in artificial urine to mimic or predict the effect of the medium on the bilayer stability after intravesical in vivo administration. No significant changes of the collected parameters were observed for both samples (empty and loaded vesicles) when prepared in Hepes buffer with respect to AUM.

**Table 2:** Bilayer features. (SD values are all in the range ± 0.01–0.02) in Hepes Buffer and in artificial urine medium. Data was obtained as the means of three independent experiments.

Sample	I_1_/I_3_ (Polarity)		I_E_/I_3_ (Microviscosity)		Anisotropy A.U. (Fluidity)	
	Hepes buffer	AUM	Hepes buffer	AUM	Hepes buffer	AUM
Nio	0.95	0.99	2.48	2.59	0.25	0.26
GM-Nio	1.24	1.24	0.84	0.99	0.34	0.35

**Figure 2:**
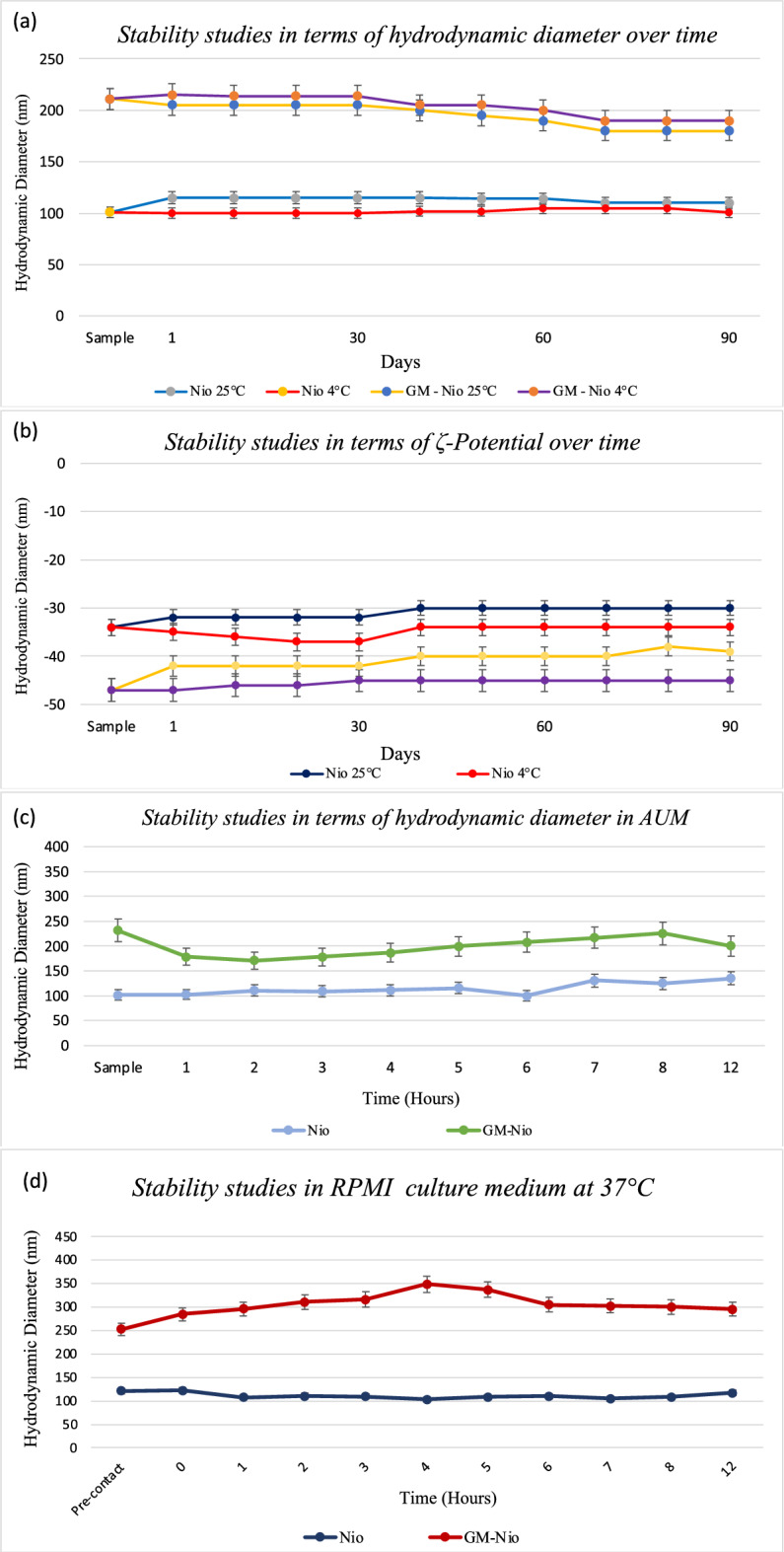
Result of investigation on physicochemical stability over time. Effect of storage temperature (25 °C and 4 °C) hydrodynamic diameter and ζ-potential (**A**, **B**) and stability studies in terms of hydrodynamic diameter in AUM (**C**) and in RPMI culture medium (**D**). Data was obtained as the means of three independent experiments.

Stability studies were performed at two different temperatures, room temperature (25 °C) and 4 °C, for both samples: loaded and empty niosomes. The data obtained showed that empty and GM-Nio formulations maintained the same size range for 3 months at the experimental conditions. From Fig. 2 panel B it is possible to affirm that no significant variations of ζ-Potential were observed for both samples at all experimental conditions texted.

Stability studies were also carried out to evaluate the empty and loaded Nio resistance to artificial urine and the culture medium employed in biological studies. By Fig. 2, panel C and D, it is possible to observe that the hydrodynamic diameter and ζ-potential have not undergone substantial changes during the experiment carried out at 37 °C for 12 h and these results do not suggest the nanocarriers degradation.

**Figure 3:**
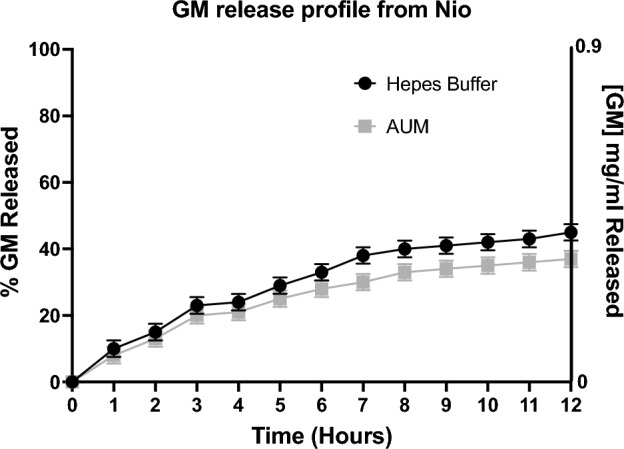
GM release profile over 12 h. Data was obtained as the means of three independent experiments.

Moreover, the culture medium (RPMI) doesn’t affect the nanocarrier hydrodynamic diameter (evaluated for 48 h, at 37 °C, see Fig. S1). Moreover, release studies were carried out to evaluate the amount of the released drug. From Fig. 3 it is possible to see that the percentage of the GM released was around 40% for both samples (GM-Nio in Hepes buffer/AUM) at 37 °C until up to 12 h (see S2 for 48 h).

### Determination of minimum inhibitory concentration (MIC) of Nio and GM-Nio formulations

**Figure 4:**
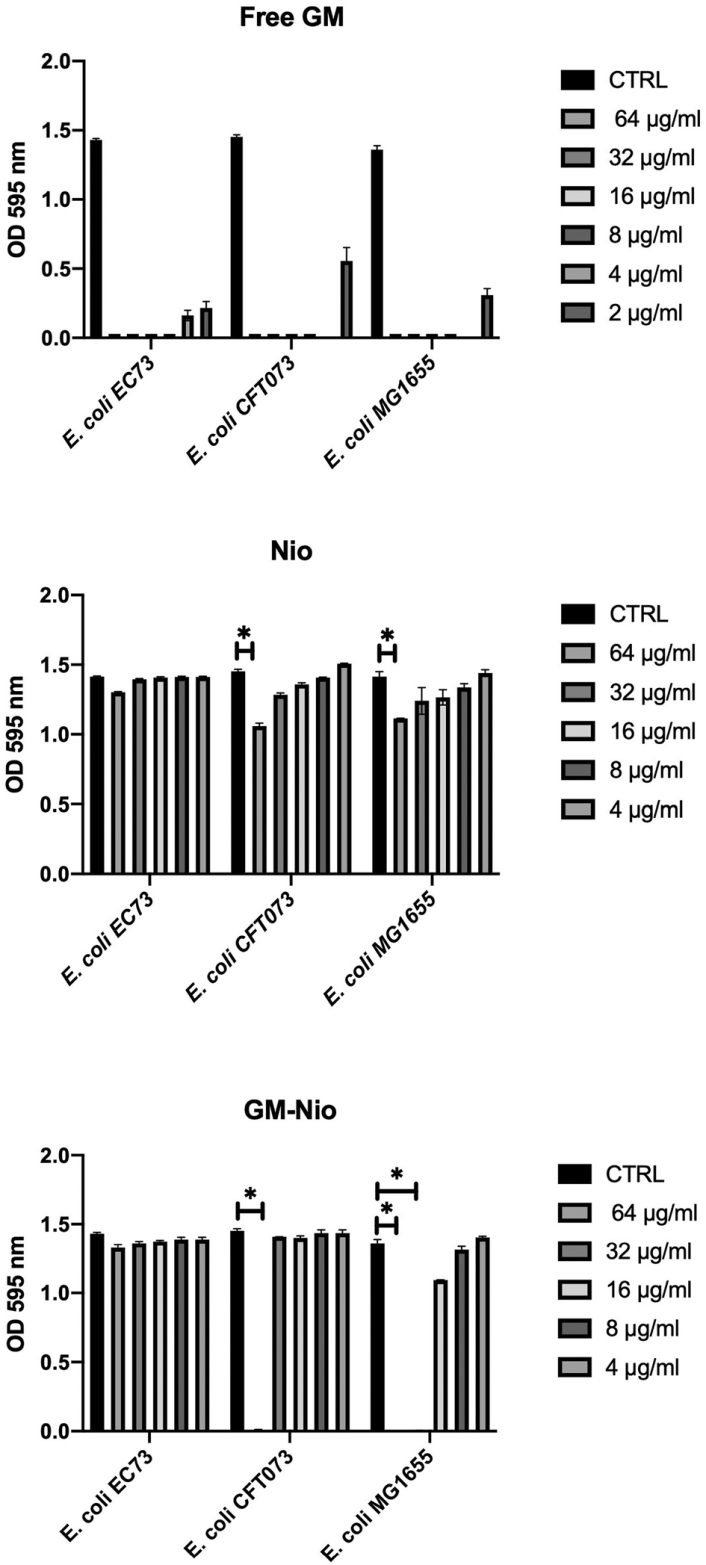
Susceptibility test with Free-GM, empty Nio and GM loaded Nio. Data were expressed as mean ± SD. All considered conditions were compared to untreated control. *p value ≤ 0.05. Data are the averages of triplicate samples from three identical experiments and the error bars represent standard deviations. Statistically significant differences compared with the negative control (untreated cells) are indicated by asterisks (*, *p* < 0.05).

Free GM, Nio and GM Nio MICs were determined by the broth microdilution method. The growth of EC73 strain was inhibited by 8 μg/ml of free antibiotic, differently to CFT073 and MG1655 strains that showed a MIC value of 4 μg/ml. For the CFT073 strain, GM-Nio showed a MIC value of about 64 μg/ml, a value that was unable to inhibit bacterial growth when microorganism was treated with Nio. All tested concentrations of both Nio and GM-Nio did not induce growth inhibition of EC73 strain. GM-Nio produced a more pronounced antibacterial effect on the MG1655 strain, in which the MIC value was about 32 μg/ml (Fig. 4).

### Nio and GM-Nio cytotoxicity

**Figure 5:**
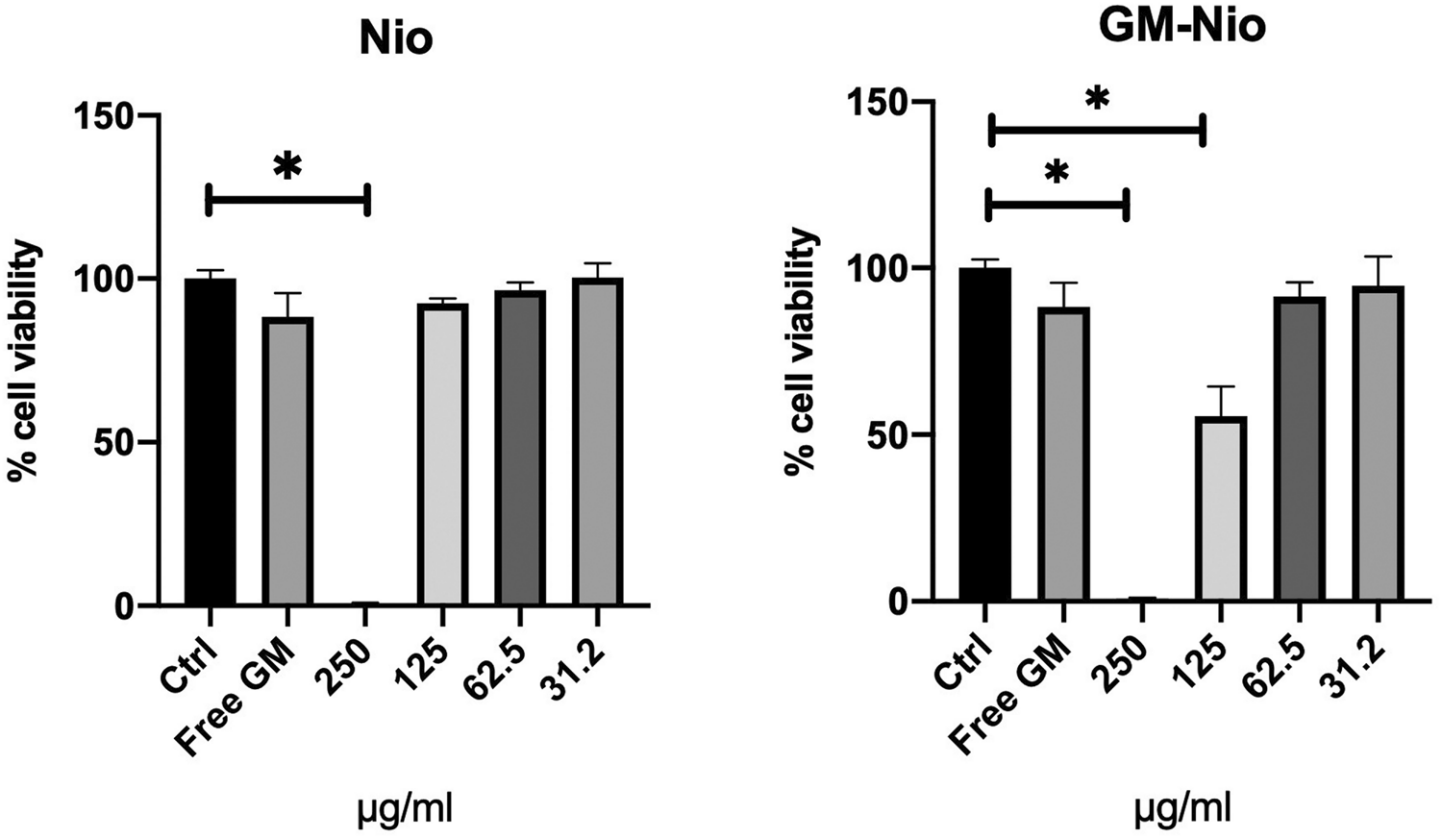
Cytotoxicity activity of empty Nio and GM loaded Nio on T24 cells. Results were expressed as % cell viability compared to untreated control. Free GM was assayed at 250 µg/ml. Data are the averages of duplicate samples from three identical experiments and the error bars represent standard deviations. Statistically significant differences compared with the negative control (untreated cells) are indicated by asterisks (*, *p* < 0.05).

The growth inhibitory activity of Nio and GM-Nio against T24 cells was assessed by colorimetric 3-(4,5-dimethylthiazol-2-yl)-2,5-diphenyltetrazolium bromide (MTT) reduction assay. For this purpose, free GM and niosomal preparations were put in contact with bladder cell monolayers and cytotoxic effects evaluated after 24 h. As shown in Fig. 5, free GM, tested at 250 µg/ml, showed no significant cytotoxicity. On the contrary, treatment with both empty and loaded niosomes, at the same concentration, resulted in a complete inhibition of cell proliferation. Furthermore, when noisomes were loaded with 125 µg/ml of GM about a 50% vitality decrease was observed compared to both empty Nio treated and control cells.

### Niosomal T24 cell interaction

**Figure 6:**
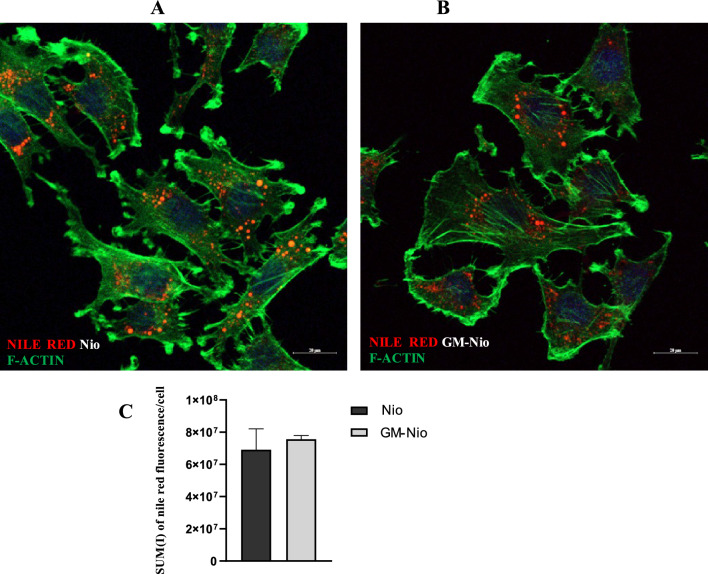
Representative confocal images of NR (**A**) and NR-GM-co-loaded niosomes (**B**) treated cells. Under these conditions nile red fluorescence is appreciable within the cell with a similar distribution pattern. Scale bars 20 µm. (**C**) The graph represents the quantitative analysis of red fluorescence intensity (Sum(I)/cell) in T24 cells treated with NR empty niosome and NR GM-loaded niosomes (means S.E.M.; Student’s t-test, *p* = n.s., n = 354 vs. 328 cells respectively) FITC phalloidin for F-actin cytoskeletal visualization was used.

Confocal analysis of Nio and GM and Nile Red co-loaded Nio (NR-GM-Nio) treated cells colored with FITC phalloidin for F-actin cytoskeletal visualization was carried out on optical spatial series with a step size of 1 µm. The analysis reveals the intracellular presence of the red labeled niosomes with peripheral and perinuclear localization (Fig. 6 panel A and B). Quantitative analysis of NR fluorescence intensity (SUM (I)) performed on optical spatial series shows that there is no significant difference between Nio and GM-Nio treated cells (*p* = n.s., n = 354 vs. 328 cells respectively).

### Effect of Nio and GM-Nio on UPEC adhesion and invasion ability

**Figure 7:**
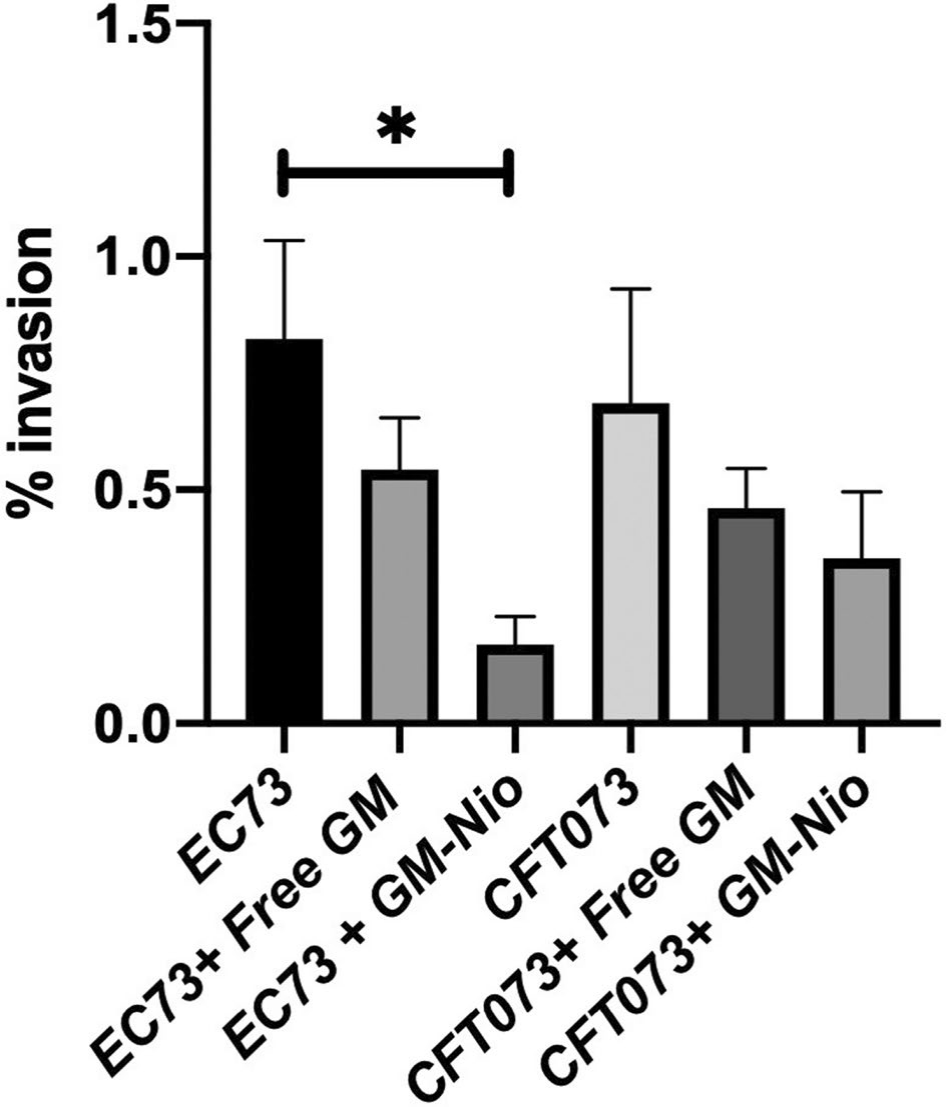
Bacterial counts and invasion percentages of T24 cells challenged with EC73 and CFT073 strains in absence or presence of GM-Nio and free GM. Results were expressed as percentage of cell invasion compared to untreated control (without niosomes). Data are the averages of duplicate samples from three identical experiments and the error bars represent standard deviations. Statistically significant differences compared with the negative control (untreated cells) are indicated by asterisks (*, *p* < 0.05).

Based on the cytotoxicity results and GM release from niosomes, we chose to assay in all experiments the concentration of 50 µg/ml for loaded niosomes. Empty niosomes were prepared using the same dilutions of loaded ones. To determine the effect of empty and GM-Nio on early steps of cell-bacteria interaction, the monolayers were treated with free GM and different niosome preparations, and bacterial adhesion to T24 cells was evaluated by colony forming unit (CFU) counts. The results obtained indicated that the percentage of adhesiveness of the reference strain CFT073 and *E. coli* EC73 strain to bladder cells was 5.4% ± 0.6 *vs* 2.8% ± 1.2, respectively. When the infected monolayers were treated with Nio or GM-Nio no significant differences on adhesion ability were observed compared to niosome untreated cells (data not shown). UPEC strains invasion ability on bladder epithelial cells was assessed by quantifying the number of intracellular bacteria after lysis of monolayers using GM protection assay. CFT073 reference strain confirmed, as already demonstrated^27^, the capacity to penetrate T24 cells; whereas EC73 strain, which has been already shown to invade prostate cells^28^, has been revealed to be able to enter also bladder cells efficiently. Invasion assay results suggested that 50 µg/ml of GM-Nio formulation, added to the monolayers during the infection period, significantly inhibited the invasion of EC73 strain, resulting in about one logarithm decrease in bacterial counts (Fig. 7).

### Detection of GFP-UPEC on T24 cells in presence of NR and NR-GM-Nio by epifluorescence

**Figure 8:**
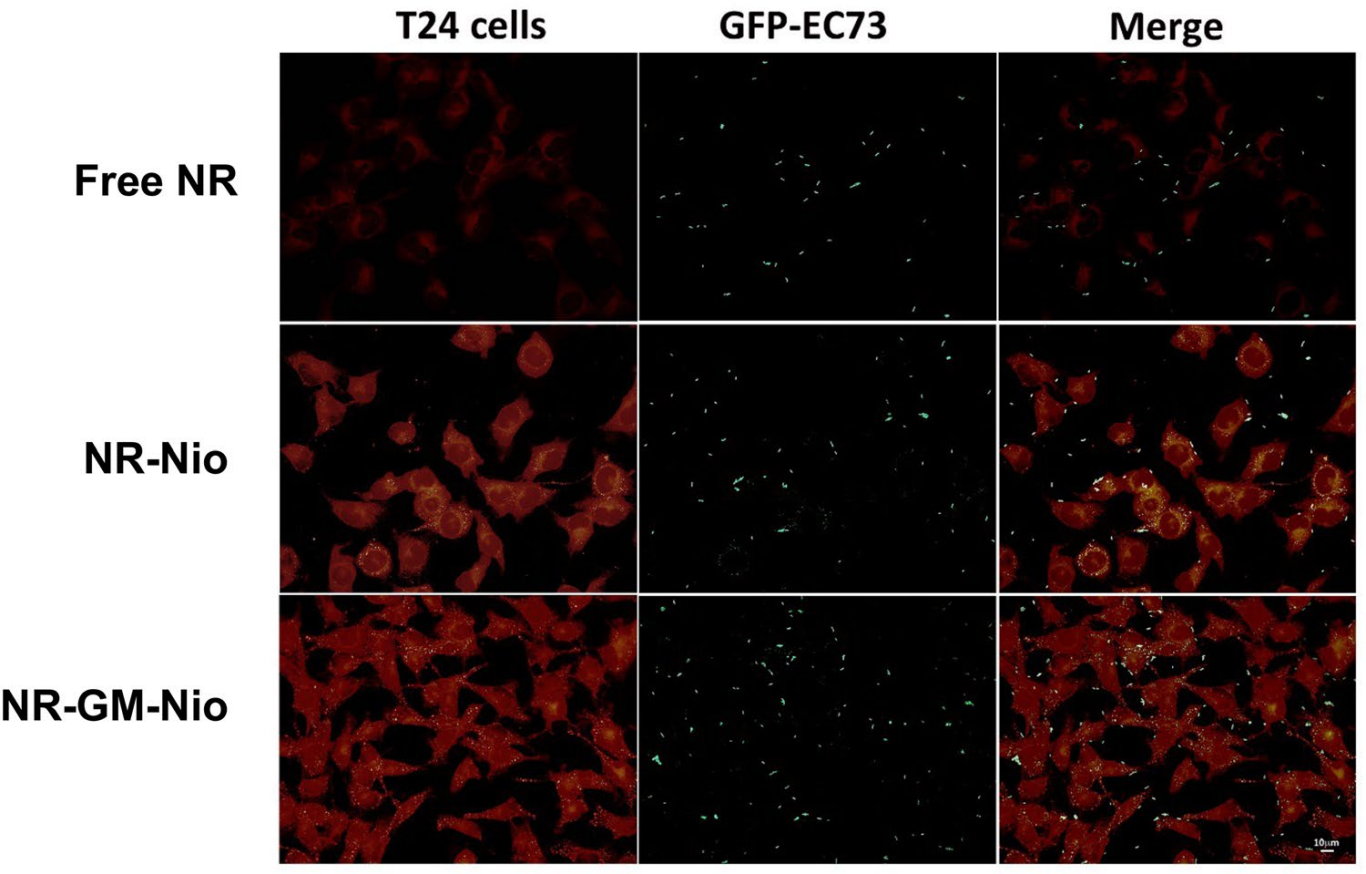
Localization of GFP-labeled *E. coli* EC73 on T24 cells during adhesion assay (1 h infection) in presence of free NR, NR- and NR-GM-niosomes.

To add further data on the interaction of UPEC strains and niosomes with bladder cells we visualized the early step of cell-bacteria interaction in presence of empty or GM niosomes loaded with NR by using GFP-EC73 (Fig. 8). This strain was chosen because of its higher invasive ability with respect to the other strains. We recorded early bacterial-cell interactions whereas the invasion step was investigated by immunofluorescence in confocal microscopy. In adhesion assay (after 1 h interaction) GFP-bacteria appeared to adhere to cells, mainly at basolateral edges, but neither apparent difference between free NR and NR-Nio or NR-GM-Nio treated cells nor colocalization with empty or GM-loaded niosomes was detected.

### Immunofluorescence analysis of intracellular bacteria

**Figure 9:**
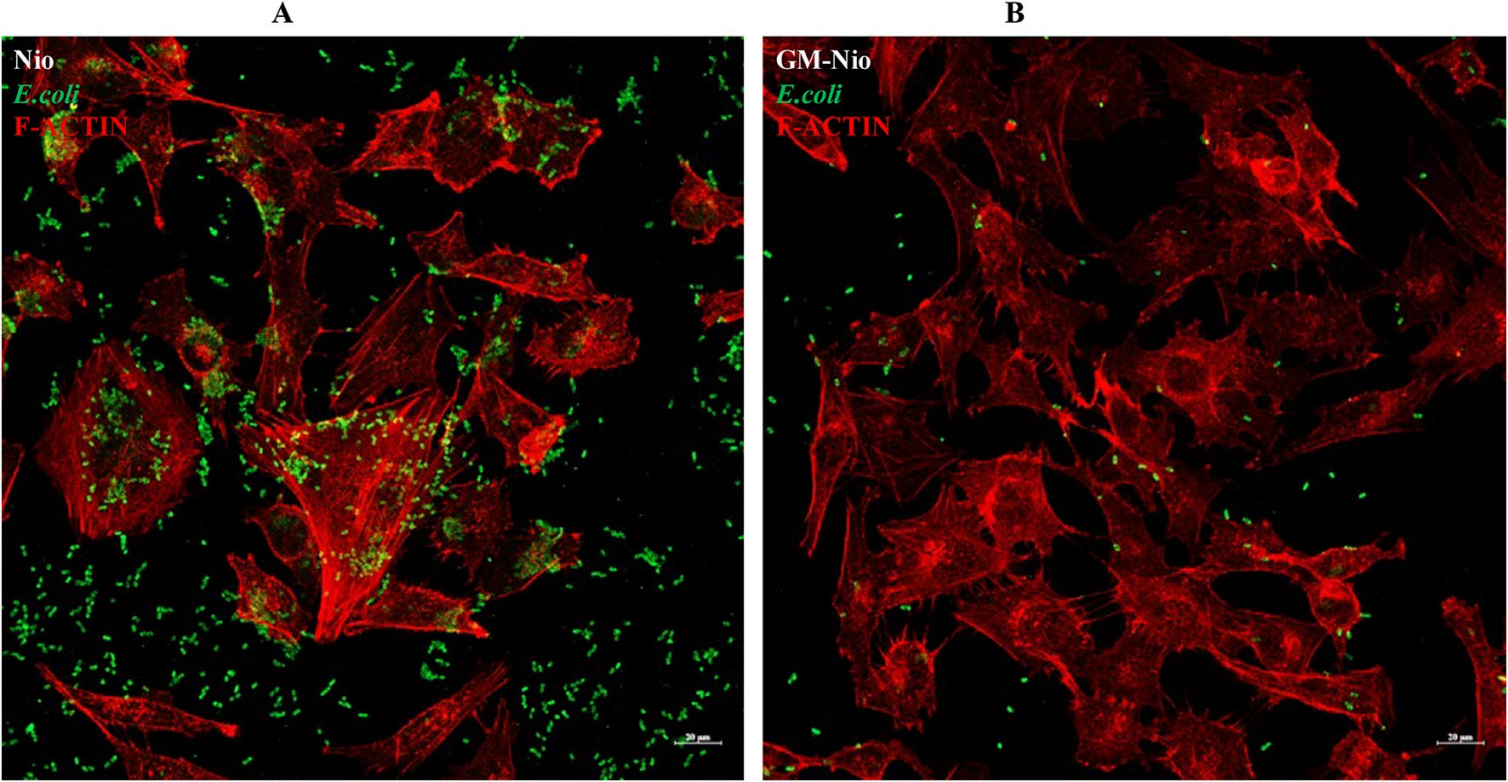
Immunofluorescence analysis of the bacteria infected cells treated with Nio (**A**) and with GM-Nio (**B**). It is evident the presence of intracellular bacteria and a clear reduction in the number of bacteria in the samples treated with GM Nio. Rodamine phalloidin for F-actin cytoskeletal visualization was used.

Immunofluorescence analysis of the bacteria was carried out both in infected cells treated with niosomes alone and with GM niosomes and colored with rhodamine phalloidin for F-actin cytoskeletal visualization. The confocal microscope analysis carried out using optical spatial series with step size of 1 µm demonstrates the presence of intracellular bacteria (Fig. 9 panel A and B). A clear reduction in the number of bacteria is evident in the samples treated with GM niosomes (Fig. 9 panel B). See also Fig. S3, panel A and B.

## Discussion

UPEC strains are the primary cause of UTIs, which either float freely in the urine and wash away when the bladder empties or form bacterial communities inside the cells that the bladder struggles to eliminate. It has been suggested that recurrent urinary tract infections are linked to the UPEC persistence and their re-emergence within the urinary tract^29^. The intracellular environment protects the pathogens against the antibiotics because of poor drug penetration. Moreover, efficacy of antibiotics such as GM, decreases in acidic organelles such as lysosomes^30^. In order to improve therapy against UTIs and prevent recurrent infections, antibacterial niosomal formulations were designed, prepared and characterized, obtaining stable nanocarriers with the ability to load and release GM, to rapidly enter bladder cells and exert antibacterial activity against UPEC strains.

The niosomes size is an important characteristic parameter that could affect the in vivo behavior of the nanocarrier. From Table 1 it is possible to observe that GM-Nio are characterized by an increased hydrodynamic diameter with respect to the empty ones and the obtained result suggest the GM inclusion inside the vesicles. Probably, the inclusion of the active compound leads to enlargement of space between the hydrophobic tails of the bilayer. This could be explained by the attraction of the positively charged GM with the negative surface of the nanocarriers with consequent surfactant displacement, enlargement of the tails in the bilayer and increased size vesicles^31^. Moreover, in agreement with the drug’s chemical structure, the GM lipophilic portion could be located inside the bilayer, in fact GM is known to have an affinity for lipophilic membranes^19^.

The bilayer destabilization could be suggested also by polarity, microviscosity and anisotropy studies; in fact, these parameters changed for GM-Nio with respect to empty ones (Table 2). Probably, a partial GM localization in the niosomal lipophilic compartment could explain the decreased microviscosity and the increased anisotropy values (which means increased bilayer rigidity)^32^.

In addition, by Table 2 it is possible to observe a slight polarity increase for GM-Nio with respect to empty ones. Probably, the bilayer destabilization due to GM niosomes inclusion, promotes the creation of the bilayer membrane defects that increase permeability to solute that allow a slight polarity increase^33^.

In addition, it is possible to observe by Table 1 that the PDI value is influenced by GM inclusion. A better PDI value characterizes the loaded vesicles with respect to empty ones^34^. The drug inclusion modifies the bilayer features and the PDI that becomes 0.20 (from 0.28 of empty niosomes). Probably, GM could stabilize the niosomes, which are characterized by narrow size distribution with respect to empty ones. In fact, PDI values of 0.20 and below are most commonly deemed acceptable for in vivo nanocarriers application^35^.

The GM inclusion does not affect the ζ-potential value, which is constant and negative enough to assure colloidal stability over time to the vesicles. In fact, ζ-potential value higher than − 30 mV or + 30 mV is generally considered to assure a sufficient repulsive force to attain better physical colloidal stability^36^. This data was confirmed by stability studies over time carried out at two different temperatures. In fact, it is possible to observe by Fig. 2, panel A and B, that the hydrodynamic diameter and ζ-potential values of both samples (empty and loaded niosomes) were maintained constant during the experiment with no significant variations, probably thanks to the ζ-potential values that assure a repulsive effect between the vesicles and thus hinders coalescence phenomena. The stability studies were also performed in artificial urine, to mimic the intravesical administration condition, and in RPMI employed during the experiments with the cultured cells.

From Fig. 2 panel C and D and Fig. S1, it is possible to observe that both media don’t affect the integrity of empty and loaded vesicles, it is possible to observe only an increase of the dimension, especially in RPMI media. This result could suggest only a surface nanocarrier interaction with RPMI components and not its degradation^37^.

The characterization studies were also carried out in the presence of UA, in fact, the stability of GM-Nio in this medium is critical and fundamental to their in vivo use and the obtained results (Fig. 2) suggest that UA does not affect the niosomes features. Moreover, by Table 2 it is possible to observe that the concentration of the GM entrapped was 1 mg/ml and it could be useful to obtain in vivo/in vitro therapeutic effect, taking into account that the proposal administration route represents a local treatment. The EE% obtained (10%) agrees with other studies of GM EE%^38^ Probably, the electrostatic interaction between GM with the negative surface of the nanocarriers could assure the GM inclusion in the niosomes^39^.

The GM release studies were carried out in Hepes buffer and AUM. By results shown in Fig. 3, it is possible to observe no significant difference of GM release trend between the sample in Hepes buffer or AUM medium. This result, according to data obtained from characterization studies, confirms that the simulated artificial urine does not affect the niosomes integrity or its release capability. In particular, the GM release percentage is similar in AUM and Hepes buffer and it is around 40%. Probably, the increased anisotropy values of the loaded sample (with consequent higher rigidity of the bilayer with respect to empty ones), affect the GM release amount. Anyway, the antibacterial in vitro studies indicate that the GM amount released is enough to assure the antibacterial activity.

Altogether, the niosomes characterization results suggested that formulated nanovesicles are suitable for interacting with cells. In fact, at 1 h treatment, a high fluorescence signal in the cells exposed to empty or GM-NR loaded niosomes, was detected. Intracellular fluorescence indicated NR presence inside the cytoplasm as both a punctate pattern with a prevalent perinuclear localization and a diffuse staining throughout the whole cell. GM carried by niosomes was probably internalized from cells and could interfere with bacterial entry and survival.

Our results from CFU counts strongly support the ability of GM niosomes formulation to decrease intracellular bacterial load to different extents among UPEC strains. Interestingly, a higher inhibitory activity of GM-Nio has been detected against intracellular EC73 strain compared to CFT073 strain, suggesting a different intracellular behavior.

Moreover, the inhibitory effect of niosomal GM appeared to be exerted after bacterial entry into cells, since co-localization experiments and CFU counts did not evidenced a difference between empty and GM-Nio during bacterial adhesion.

Many studies have shown the successful internalization of negative surface nanocarriers^40^. It was observed that cationic drug delivery systems commonly use the clathrin pathways, whereas anionic ones could undergo internalization via caveolae pathways^41,42^. It is possible to hypothesize that GM-Nio, characterized by a negative surface, can follow this route to bind the cell surface and enter. This hypothesis will be investigated and will be the focus of the future studies.

UPEC strains invade host bladder epithelial cells through endocytosis, and/or pinocytosis of caveolae and clathrin-coated pits assembled on the plasma membrane^43,44^. UPEC transported in caveolae and clathrin-coated pits may sort into specific pathways that determine the UPEC fate. As an example, it was reported that Afa/Dr adhesins and type 1 pili-expressing UPEC strains, by entering host cells through caveolae-like lipid raft domains, may avoid immediate fusion with lysosomes^43^. Moreover, it was reported that once caveolae are detached from plasma membranes, they fuse with a cell compartment called caveosome that exists at neutral pH. Caveosomes can bypass lysosomes and therefore protect the contents from hydrolytic enzyme and lysosomal degradation^45,46^. However, much evidence suggests that all major endocytosis pathways can deliver their contents to the early endosomes. From the early endosomes, cargo can be recycled back to the cell surface or can remain inside the early endosome as it converts to a late-endosomal compartment and is eventually trafficked to the lysosomes^47^.

The different extent of intracellular inhibition against both CFT073 and EC73 strains suggested that both bacterial structural and genotypic features and the endocytic pathways they follow to invade bladder cells are probably responsible for the different efficacy of GM-Nio. Regarding CFT073, some authors recently demonstrated that its survival within lysosomes was due to the ability to avoid acidification of the LAMP1 + compartments via *hly*A expression. The disruption of microtubule formation within infected bladder cells promotes intracellular bacterial survival, by impeding acidification of bacteria-harboring lysosomes^48^. Conversely, since EC73 strain is devoid of *hly*A gene, as reported by Conte et al.^28^ we could suppose that this strain follows a different entry pathway to infect bladder cells compared to CFT073.

Whatever the mechanism of niosomes or UPEC entry into cells, GM delivered by niosomes have to maintain its activity to exert the inhibitory effect observed in this study. Based on pH values of nearly neutrality found in caveosomes, caveolae and early endosomes and UPEC strategies to avoid endosome-lysosome fusion, it is reasonably hypothesized that intracellular delivered GM is able to maintain its activity.

T24 bladder cells, infected with intracellular UPEC, were used as a study model for efficacy testing of nanocarriers loaded with GM, that notably, is characterized by a poor entry into the cells. Notwithstanding the treatment with GM-Nio in *E. coli* EC73 infected cells induced a slight reduction (threefold) in the percentage of invasion respect to free GM, optimized GM-Nio, could represent a promising solution to reach satisfactory levels in intracellular compartments.

## Conclusion

The multidisciplinary approach employed to understand the physical–chemical features of the empty and loaded nanocarriers is fundamental to demonstrate that the designed niosomes are suitable for supposed application. In particular, in this study it has been highlighted that the GM-Nio exhibited good stability over time and in AUM (very important in view of intravesical administration), appropriate hydrodynamic diameter, EE%, and release capability. Moreover, GM-Nio showed activity against intracellular UPEC strains, they are promising in terms of enrichment of infection microenvironment target sites, and they represent a promising approach for intracellular antibiotic release. More deep and appropriate studies will be carried out to investigate how GM-Nio interacts with the cells in order to understand and explain the obtained result and predict the in vivo behavior of the loaded nanocarriers.

## Methods

### Niosomes preparation and characterization

Niosomal vesicles were prepared by Thin Layer Evaporation (TLE) technique^49^.

The components were weighed according to the quantities described in Table 3 and solubilized by means of an organic mixture consisting of chloroform:methanol 3:1 (v/v).

**Table 3:** Quali-quantitative composition of both samples.

Sample	Tween 85 (mM)	Span 80 (mM)	Gentamicin (mg/ml)
Nio	22.5	22.5	–
GM-Nio	22.5	22.5	10.0

Subsequently, the organic solvent was evaporated by subjecting the sample to the action of a Rotavapor (Büchi-Italia S.r.l., Assago (MI), Italy) at room temperature and an oil pump.

Then the sample is hydrated; in the case of “empty’’ niosomes the hydration is carried out by 5 ml of Hepes buffer (pH = 7.4, M = 0.01), while in the case of “loaded’’ niosomes are used 5 ml of Gentamicin solution (10 mg/ml).

By exploiting the mechanical energy, by means of a vortex, the separation of the thin layer of film from the walls of the test tube was facilitated and a multilamellar suspension was obtained.

An ultrasonic sonicator (Vibracell-VCX 500, Sonics, Taunton, MA, USA) was used to obtain unilamellar vesicles^50^ (5 min, 60 °C and 25% of Amplitude).

To remove from the suspension any impurity or unstructured component in the vesicular suspension, a purification of the sample was carried out in a chromatographic column, using the separation technique by size exclusion (SEC). The chromatography column is made of Sephadex G-75, a dextran polymer resin. Finally, the samples were filtered using a semipermeable membrane (MF-Millipore®, Ireland, E.U., 0.22 μm), in order to retain the impurities and ensure the sterility of the samples, in accordance with the European Pharmacopoeia^32^.

### Dynamic light scattering and ζ-potential measurements

Hydrodynamic diameter, ζ-potential and polydispersity index (PDI) of empty and loaded niosomes were evaluated, using Malvern Nano ZS90 apparatus (Malvern Instruments, Worcestershire, UK), equipped with a 5 mW HeNe laser, λ = 632.8 nm. The scattering angle was 90° and the analysis of the intensity autocorrelation function was carried out using the Contin algorithm. The calculated mean hydrodynamic radius corresponds to the intensity weighted average^51^. Electrophoretic mobility of the vesicles was measured by laser Doppler anemometry using the Malvern Zetasizer Nano ZS90 apparatus (Malvern Instruments, Worcestershire, UK). The ζ-potential was obtained by converting the mobility (u) using the Smoluchowski relation ζ = uη/ð, where η is the viscosity and the permittivity of the solvent phase^52^.

### Morphological visualization of niosomes

Morphology of empty and GM-loaded niosomes was obtained by visualizing the samples by Transmission Electron Microscopy (TEM). One drop of samples was placed into a formvar carbon-coated grid and, after adsorption, niosomes were negatively stained with 2% (v/v) filtered aqueous sodium phosphotungstate acid (PTA). A FEI 208S transmission electron microscope (FEI Company, Hillsboro, OR, USA) equipped with the Mega-view II SIS camera (Olympus) was used for visualizing and capturing images at an accelerating voltage of 100 kV. Adobe Photoshop software was used to optimize image editing.

### Fluorometric measurements

Characterization studies on niosome bilayer were conducted using two fluorescent probes: DPH and pyrene. Information was obtained regarding the microviscosity, polarity and fluidity of the membrane^53^. The samples loaded with DPH were prepared by codissolution of surfactants and DPH solution (2 × 10^−4^ M) in the organic mixture, consisting of methanol/chloroform, then the same preparation method as described for the samples was followed.

DPH fluorescent measurements were performed with excitation λexc = 350 nm and detecting the fluorescence intensity at λem = 428 nm, using a luminescence spectrometer (LS5013, PerkinElmer, Waltham, MA, USA).

The fluorescence anisotropy (r) was determined by using Eq. (1):

1 $$Fluorescence\;anisotropy\left( r \right) = \frac{{\left( {I_{vv} - I_{vh} } \right) \times G}}{{\left( {I_{vv} - 2I_{vh} } \right) \times G}}$$

where I_VV_, I_VH_, I_HV_, and I_HH_ are fluorescent intensities, and subscript V (vertical) and H (horizontal) represent the orientation of polarized light and G = I_HV_/I_HH_ factor is the ratio of sensitivity of detection system for vertically and horizontally polarized light.

Additionally, studies were performed utilizing a fluorescent probe: the pyrene. The samples were prepared by adding the probe at a concentration of 4 mm to the components and the same preparation method as described above was followed.

This fluorescent probe is characterized by a spectrum characterized by five emission bands (I_1_-I_5_) as monomer and one as excimer (I_E_). The intensities of these peaks depend on the mobility and lateral distribution of the probe within the oily phase.

The signals emitted by the sample were recorded with an emission spectrum with λ = 350–550 nm and Ex = 330 nm. The relationship between the different intensities provides indications regarding the microviscosity and the polarity. In particular, the I_1_/I_3_ ratio, corresponding to the first and third vibration bands of the Pyrene spectrum, is linked to the polarity of the probe environment. While the I_E_/I_3_ ratio characterizes the microviscosity of the nanosystem; in fact, depending on the viscosity, the pyrene can organize itself and form the intramolecular excimer^54^.

### Physico-chemical Stability

Several stability studies were performed on both the empty and loaded niosomes. These experiments, conducted using DLS (Malvern Instruments, Worcestershire, UK), consist of monitoring over time the changes in size and ζ-potential. The stability of the samples was initially evaluated for 90 days at room temperature and 4 °C. As a preliminary biological evaluation, stability in Artificial Urine Medium (AUM) and RPMI culture medium were then evaluated for 12 h. Artificial urine, at pH: 6.6, was prepared according to Monika Pietrzyn ´ska et al.^55^, and the composition is reported in Table 4.

**Table 4:** Chemical composition of artificial body urine (pH 6.6).

Reagent	Dosage (g)
Urea	25.0
NaCl	9.0
NH_4_CL	3.0
Creatinine	2.0
Na_2_HPO_4_	2.5
KH_2_PO_4_	2.5
Na_2_SO_3_	3.0
Distilled water	Total 1.0 L

To carry out these experiments, 1 ml of sample was added to 1 ml of AUM or RPMI and placed in a test tube, subject to a magnetic stirrer. Measurements are carried out at different time intervals (every hour for twelve hours), in terms of particle size and ζ-potential.

### In vitro release studies

In vitro drug release studies were conducted by placing 1 ml of the sample (GM-Nio) and 1 ml of the Hepes Buffer or AUM inside a dialysis tube (molecular weight cut-off: 8000 MW by Spectra/Por®). Dialysis tubes were immersed in Hepes Buffer (10 mM, pH 7.4) placed in magnetic agitation and at 37 °C throughout the experiment.

Drug concentration released by niosome was measured by UV spectrophotometer (Lambda 25, PerkinElmer, Waltham, MA, USA). Aliquots of 1 ml of external phase (Hepes Buffer) were taken and measured, by UV-analysis, every hour for 48 h and then re-inserted back in the external medium.

### Bacterial strains

Uropathogenic *Escherichia coli* strain EC73 were from a patient with recurrent UTIs^28^ *E. coli* ATCC 700928 (CFT073)^56^ (ATCC, Manassas, VA, USA) and non-invasive *E. coli* K12 MG1655 were used as reference strains. Bacterial strains were grown in Brain Heart Infusion Broth (BHI) (Oxoid, Basingstoke Hampshire, UK) and maintained as stock cultures in 15% glycerol-BHI at − 80 °C.

### GFP plasmid transformed strains

*Escherichia coli* strains were transformed with the GFP‐expressing plasmid pFPV25.1 as described by Valdivia and Falkow^57^ using the calcium chloride method. Transconjugants were selected on LB agar plates with ampicillin (100 µg/ml) overnight at 37 °C. Green fluorescent transconjugant colonies were picked and stored in glycerol at − 80 °C.

### Determination of minimum inhibitory concentration (MIC) and minimum bactericidal concentration (MBC) of GM

Free GM, Nio and GM Nio MICs were determined by the broth microdilution method and carried out in triplicate. Exponentially growing bacterial cultures were diluted to the cell density corresponding to 0.5 McFarland, and 10 μl of each bacterial suspension was added to 190 µl of BHI (Oxoid, Basingstoke Hampshire, UK) containing the drug concentrations ranged from 125 to 7.8 µg/ml. After the incubation at 37 °C for 24 h, the bacterial growth was evaluated by measuring the optical density (OD) at 595 nm and MIC was defined as the lowest concentration of the drug at which bacterial growth was inhibited. MBC was determined by sub-culturing on Tryptic Soy Agar (TSA, Oxoid, Basingstoke Hampshire, UK),for 24 h, 10 μL from each well with no visible growth. MBC is defined as the lowest drug concentration that kills 99.9% or more of the bacterial inoculum.

### Cell line

The human bladder cancer cell line (T24) was obtained from the American Type Culture Collection (Manassas, VA, USA). Cells were cultured in Roswell Park Memorial Institute (RPMI) 1640 medium supplemented with 10% heat-inactivated fetal bovine serum (FBS; SAFC Biosciences Inc., Lenexa, KS, USA), 100 IU/L penicillin and 100 mg/L streptomycin. Cultures were maintained in a humidified atmosphere containing 5% CO_2_ at 37 °C.

### Cytotoxicity studies by MTT assay

T24 cells at the concentration of 5X10^5 cells/ml were seeded in 96-well plates and cultured for 24 h at 37 °C with 5% CO_2_. Different concentrations of Nio and GM-Nio, starting from 250 to 31.2 µg/ml were added to cell monolayers and incubated for 24 h. Empty niosomes were prepared using the same dilutions as performed with antibiotic-loaded niosomes. Then, 100 μL of 0.5 mg/ml of 3-(4,5-dimethylthiazol-2-yl)-2,5-diphenyltetrazolium bromide (MTT) reagent was added to each well and plates were incubated at 37 °C for an additional 4 h. Afterwards the dye was eluted with 200 μL of DMSO for 10 min at room temperature and, finally, the optical density at 568 nm was measured using a microplate reader (PerkinElmer, Boston, MA, USA). The viability percentage (%) was calculated as: Mean OD treatment/Mean OD control × 100. Free GM was also tested at 250 μg/ml as comparison.

### Confocal analysis for niosome localization

Localization of niosomes inside T24 cells was obtained by confocal microscopy. T24 cells were cultured into 8-well µ-slides and treated with Nio and GM-Nio. At the end of incubation, the cells were fixed with 4% paraformaldehyde for 10 min at 4 °C and washed twice for 10 min with PBS. DAPI fluorescent dye (Invitrogen Molecular Probes Eugene, USA, 1:400 dilution) for nuclei staining and FITC phalloidin (Invitrogen Molecular Probes, Eugene, USA 1:40 dilution) for F-actin visualization were utilized. The slides were then mounted with 0.1 mM Tris–HCl at pH 9.5: glycerol (2:3) and analyzed with Zeiss LM900 confocal microscope. The images were scanned under a 20X objective. Optical spatial series with a step size of 1 µm were recovered. Laser intensities were maintained fixed during the acquisition of the different samples. The intensity of the red fluorescence was determined with the use of Zen 3.0 Blue Edition software, using the sum of intensity (Sum(I)). The quantitative data obtained by orthogonal projections were normalized for the number of cells recovered (354 vs 328 in Nio and GM-Nio treated cells respectively) and means ± S.E.M. was graphed.

### Adhesion assay

Adhesiveness of *E. coli* strains to T24 monolayers was assayed by culturing the cells in a 24‐well plate at a density of 2 × 10^5 cells/ml for 24 h at 37 °C in 5% CO_2_. T24 cells, infected with *E. coli* strains at a multiplicity of infection (MOI) of approximately 10 bacteria per cell, were centrifuged twice at 500 g for 2.5 min to synchronize infection and incubated for 30 min at 37 °C in 5% CO_2_^58^. After the incubation time, cells were extensively washed with PBS to remove unattached bacteria, lysed adding ice‐cold 0.1% Triton X‐100 and seeded on TSA plates. After 24 h of incubation at 37 °C, bacteria were counted and the adherence was considered when the mean adhesion index (number of adherent bacteria/initial inoculum) was ≥ 0.8%. To determine antibacterial GM-Nio effect, adhesion ability was also evaluated by culturing T24 cell monolayers in the same conditions in presence of different niosomes formulations.

### Invasion assay

Bacterial invasion of bladder epithelial cells was evaluated by GM protection assays as previously described^28^. Briefly, T24 cells, cultured to confluence in 24-well plates, were inoculated with a MOI of 10 bacteria per host cell in duplicate wells and then centrifuged twice at 500 g for 2.5 min to synchronize infection. After the centrifugation, monolayers were incubated for 1 h at 37 °C in 5% CO_2_. Monolayers were then washed with PBS and incubated for 60 min in a growth medium containing gentamicin (100 µg/ml) (Thermo Fisher Scientific, Waltham, MA, USA) (conditions under which gentamicin is membrane impermeative and almost exclusively kills extracellular bacteria). After washing, cells were lysed with a solution of 1% vol/vol Triton X-100 and dilutions of cell lysates were plated on TSA plates to determine the number of viable bacteria. A strain was considered invasive when the ratio between the number of intracellular bacteria and the initial inoculum was 0.1%. The assay was performed in triplicate. *Escherichia coli* MG1655 was used as negative control.

To verify the putative inhibitory effect of empty Nio and GM-Nio on intracellular *E. coli s*trains in the infected T24 cells, 50 µg/ml of niosomes were put in contact with the bladder monolayers during the infection period. After eliminating the extracellular bacteria by gentamicin (100 µg/ml), the number of intracellular bacteria was determined as described above.

### Bacterial interaction with T24 cells in presence of niosomes by epifluorescence microscopy

In order to study early niosome-bacteria interactions, T24 cells were seeded on 8-well chamber-slides (Falcon, Corning, NY, USA) for 24 h at 37 °C and exposed to GFP-*E.coli* EC073 strain in presence of niosomes loaded with Nile Red dye (a hydrophobic fluorophore dye molecule) or GM-NR co-loaded niosomes for 1 h at 37 °C. After extensive washing with PBS, all different treated samples were fixed in methanol/acetone (1:1) for 5 min at − 20 °C and slides mounted with 0.1% (w/v) p-phenylenediamine in 10% (v/v) PBS, 90% (v/v) glycerol, pH 8.0. Fluorescent images were taken using a Leica DM4000 fluorescence microscope (Leica Microsystem, Wetzlar, Germany) equipped with an FX 340 digital camera and processed with Adobe Photoshop CS4 software (Adobe Systems, San Jose, CA, USA). A 1 mg/mL stock solution of NR was used at a final concentration of 100 ng/mL to treat cells as free dye either in association or not with bacteria.

### Immunofluorescence analysis of bacteria

Immunofluorescence analysis of bacteria was performed in semi confluent cells, cultured into 8-well µ-slides (ibidi GmbH, AmKlopferspitz19, D-82152 Martinsried, Germany). T-24 cell monolayers were infected with *E. coli* strains and during the infection time cells were treated with Nio and GM-Nio. At the end of incubation, the cells were fixed with 4% paraformaldehyde for 10 min at 4 °C and washed twice for 10 min with PBS. The cells were permeabilized for 1 h using PBS, 1% BSA (Bovine Serum Albumin, Santa Cruz Biotechnology), 0.1% Triton X-100 (Sigma-Aldrich), followed by the incubation overnight at 4 °C with goat anti-*E. coli* antibody (Bio-Rad, Berkeley, USA). The cells were washed with PBS and incubated for 45 min at room temperature with the secondary antibody (rabbit anti-goat Alexa Fluor 488, green, diluted 1:500; Invitrogen, Waltham, USA). DAPI fluorescent dye (Invitrogen Molecular Probes Eugene, USA, 1:400 dilution) for nuclei staining and rhodamine phalloidin (Invitrogen Molecular Probes Eugene, USA 1:40 dilution) for F-actin visualization were utilized. The slides were then washed with PBS and mounted with 0.1 mM Tris–HCl at pH 9.5: glycerol (2:3). Negative controls were processed in the same conditions besides primary antibody staining. Finally, immunolocalization was analyzed with Zeiss LM900 confocal microscope. The images were scanned under a 20X objective. Optical spatial series with a step size of 1 µm were recovered.

### Statistical analysis

One-way and repeated-measures analyses of variance (ANOVA) followed by post hoc Student's unpaired and paired t tests, as needed, were used to assess statistical significance. In all cases, a *P* value of ≤ 0.05 was considered statistically significant.

### Supplementary Information

Supplementary Figures.

## Data Availability

The datasets used and/or analyzed during the current study available from the corresponding author on reasonable request.
