# Supplementary material for: Gentamicin loaded niosomes against intracellular uropathogenic Escherichia coli strains
Source: Sci Rep. 2024 May 3;14:10196. doi: 10.1038/s41598-024-59144-x (PMC11068731; doi:10.1038/s41598-024-59144-x)
Supplement: Supplementary file 1 — Supplementary Figures. [file 41598_2024_59144_MOESM1_ESM.docx]

**Supplementary information:**

**
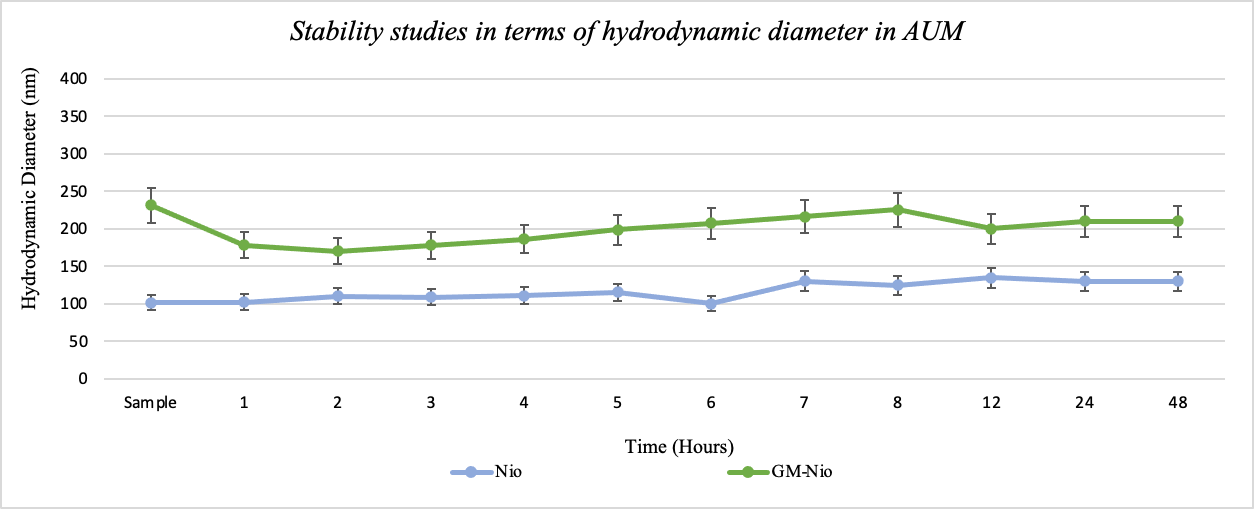
**

(a)


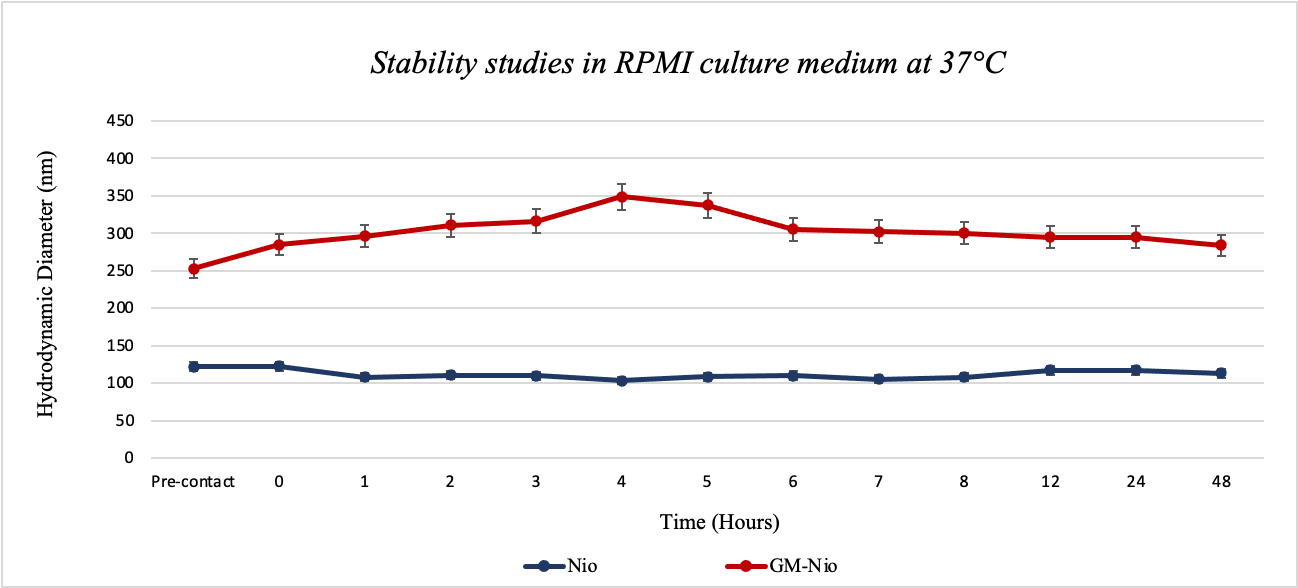


(b)

**Figure S1** Stability studies in terms of hydrodynamic diameter in AUM (a) and in RPMI culture medium (b).


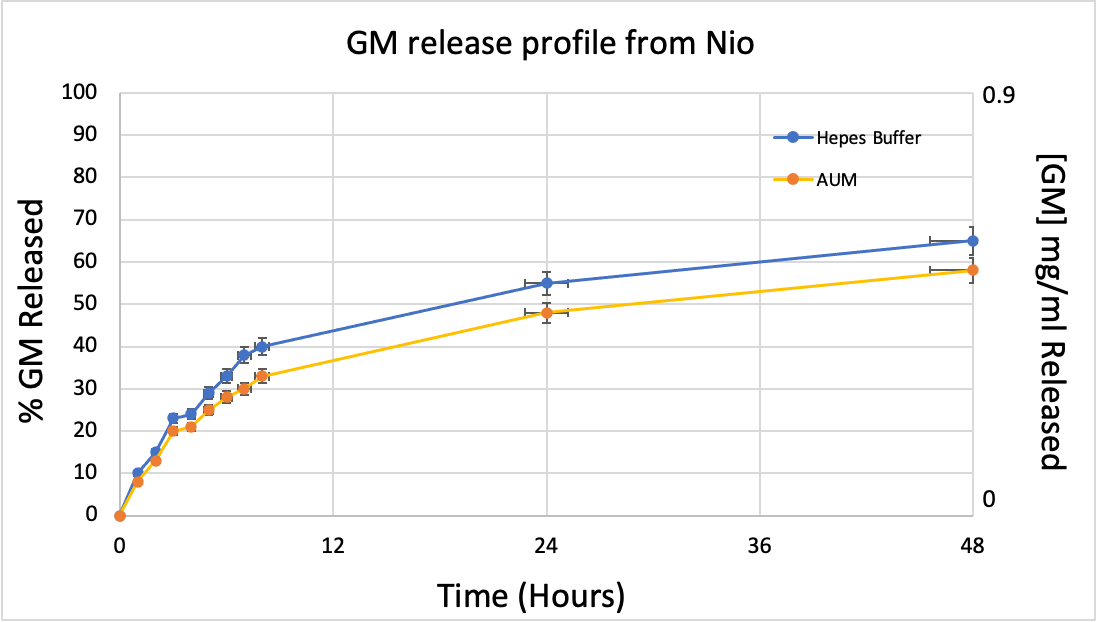


**Figure S2.** Release profile of Gentamicin over 12 hours. Data were obtained as the mean of three independent experiments.


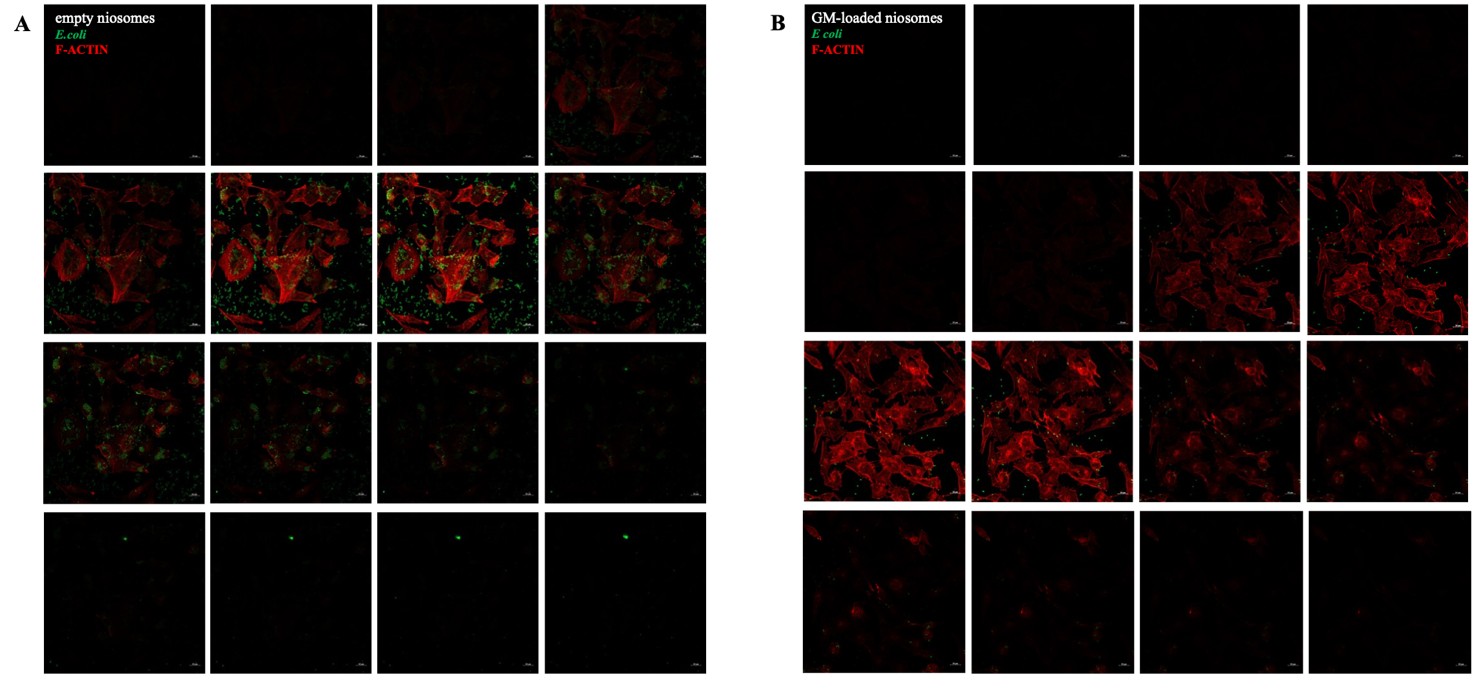


**Figure S3.** Panel A: Z-stack profile of bacteria (green) infected cells treated with Nio. Rodamine phalloidin for F-actin cytoskeletal visualization was used. Panel B: Z-stack profile of bacteria(green) infected cells treated with GM-Nio. Rodamine phalloidin for F-actin cytoskeletal visualization was used.
